# Temperature and preeclampsia: Epidemiological evidence that perturbation in maternal heat homeostasis affects pregnancy outcome

**DOI:** 10.1371/journal.pone.0232877

**Published:** 2020-05-18

**Authors:** Sagi Shashar, Itai Kloog, Offer Erez, Alexandra Shtein, Maayan Yitshak-Sade, Batia Sarov, Lena Novack

**Affiliations:** 1 Clinical Research Center, Soroka University Medical Center, Ben-Gurion University of the Negev, Beersheba, Israel; 2 Department of Geography and Environmental Development, Faculty of Humanities and Social Sciences, Ben Gurion University, Beersheba, Israel; 3 Division of Obstetrics and Gynecology, Maternity Department "D", Soroka University Medical Center, Ben-Gurion University of the Negev, Beersheba, Israel; 4 Department of Environmental Health Exposure, Epidemiology, and Risk Program Harvard T.H. Chan School of Public Health, Boston, Massachusetts, United States of America; 5 Department of Public Health, Faculty of Heath Sciences, Ben-Gurion University of the Negev, Beersheba, Israel; University of Mississippi Medical Center, UNITED STATES

## Abstract

**Introduction:**

This study aims to determine the association between temperature and preeclampsia and whether it is affected by seasonality and rural/urban lifestyle.

**Methods:**

This cohort study included women who delivered at our medical center from 2004 to 2013 (31,101 women, 64,566 deliveries). Temperature values were obtained from a spatiotemporally resolved estimation model performing predictions at a 1×1km spatial resolution. In “Warm” pregnancies >50% of gestation occurred during the spring-summer period. In cold pregnancies >50% of gestation occurred during the fall and winter. Generalized estimating equation multivariable models were used to estimate the association between temperature and incidence of preeclampsia.

**Results:**

1) The incidence of preeclampsia in at least one pregnancy was 7% (2173/64,566); 2) during “warm” pregnancies, an elevation of one IQR of the average temperature in the 1^st^ or the 3^rd^ trimesters was associated with an increased risk to develop preeclampsia [patients with Jewish ethnicity: 1^st^ trimester: relative risk (RR) of 2.38(95%CI 1.50; 3.80), 3^rd^ trimester 1.94(95%CI 1.34;2.81); Bedouins: 1^st^ trimester: RR = 2.91(95%CI 1.98;4.28), 3^rd^ trimester: RR = 2.37(95%CI 1.75;3.20)]; 3) In “cold” pregnancies, an elevation of one IQR of average temperature was associated with a lower risk to develop preeclampsia among patients with Bedouin-Arab ethnicity RR = 0.68 (95% CI 0.49–0.94) for 1^st^ trimester and RR = 0.62 (95% CI 0.44–0.87) for 3^rd^ trimester.

**Conclusions:**

1) Elevated averaged temperature during the 1^st^ or 3^rd^ trimesters in “warm” pregnancies confer an increased risk for the development of preeclampsia, especially in nomadic patients; 2) Of interest, during cold pregnancies, elevated averaged temperature was associated with a lower risk to develop preeclampsia for nomadic patients. 3) These findings suggest temperature might be associated with perturbations in maternal heat homeostasis resulting in reallocation of energy resources and their availability to the fetus that may increase the risk for preeclampsia. This observation is especially relevant in the context of global warming and its effects on maternal/fetal reproductive health.

## Introduction

Global warming is an emerging concern of our time as scientists suggest that climate and weather changes are quickly rising; temperature has risen about 0.74°C during the 21st-century [1]. Pregnant women, the developing fetus, and young children are considered the most vulnerable to the environmental effects of climate change [2]. Although hazards of increasing temperature for human health are known [3–5] its effect on pregnancy outcome are not fully recognized [2, 6].

Accumulating evidence suggested that environmental factors may play a role in the pathophysiology of preeclampsia [7–13]. Indeed, preeclampsia was more likely to occur among women who conceived during the warmer period of the year and delivered during the colder months [11, 14–17]. Because few studies assessed the association between ambient temperature and preeclampsia, the window period of heat effect remains unclear [17]. To decrease its impact on the association between temperature and preeclampsia, Auger et al [18] adjusted to seasonality. An additional parameter that may affect the association of environmental factors and preeclampsia is socioeconomic status [17] as it reflects among other things the patients’ urban/rural lifestyle. The latter group is more exposed to the effect of environmental factors and lacks the weather balancing effect of urban leaving such as air conditioning.

We have chosen to explore the association between lifestyles in the area of Southern Israel because it is comprised predominantly of urban population of Jewish ethnicity and rural population of Bedouin-Arab ethnicity. The latter group usually belongs to a low socio-economic stratum (SES) [19], and about half resides in temporary shacks or tents, with limited access to air conditioning and municipal infrastructure [20].

Therefore, the current retrospective cohort study aimed to determine whether: 1) The temperature during gestation is associated with subsequent development of preeclampsia; and 2) The effect of seasonality and urban/rural lifestyle on this association.

## Materials and methods

### Study population

We conducted a cohort study including women who delivered at the Soroka University Medical Center (SUMC) between 2004 and 2013 and met the inclusion criteria. The SUMC is the only provider of tertiary care in the region treating most of the deliveries, with minor exceptions of residents choosing remote hospitals. The study was approved by the Soroka University Medical Center Institutional Ethics Committee (0257-15-SOR).

### Inclusion and exclusion criteria

Since home addresses are obtained only of women registered in Clalit HMO, the study cohort included only these women, covering approximately 70% of the population in southern Israel. We included pregnancies of women living in Southern Israel, aged 18–50 years, their gestational length was between 24–42 weeks and infant’s weight 500–5000 grams. Women with unknown addresses were excluded as their temperature exposure could not be estimated.

### Temperature assessment

Temperature data were obtained from a spatiotemporally resolved estimation model performing predictions of daily average temperatures at a 1 × 1 KM spatial resolution developed by Rosenfeld et al [21]. In brief, we used mixed model regressions for calibrating satellite-based observations to relevant PM monitoring data collected within 1 km of a satellite reading. We also incorporated additional spatial, temporal (daily), and spatio-temporal covariates as predictors in this calibration stage. In the second stage, we used the model fits from the 1^st^ stage to estimate temperatures in grid cells without monitors but with available satellite measurements. Finally, we used inverse distance weighted (IDW) interpolations and thin plate splines (using a smooth nonparametric function of longitude and latitude) to fill in neighboring cells without surface monitors or missing temperatures. To robustly check model performance, we used rigorous Out-of-sample ten-fold cross validation (CV) to quantify the accuracy of our predictions. Our model performance was excellent for both days with and without available satellite surface temperature observations for both Aqua and Terra (CV Aqua R2 results for min 0.966, mean 0.986, and max 0.967; CV Terra R2 results for min 0.965, mean 0.987, and max 0.968). Our validation showed that daily min, mean and max air temperature can be reliably used as robust exposure assessment using daily MODIS satellite surface temperature data across Israel [22].

Daily temperatures in pregnancy were assigned to each mother based on her residential address after been geocoded by ArcGIS version 10.4.1 (ESRI, 2018). The temperature estimates were further averaged over each trimester.

### Seasonality

Seasons were defined according to Alpert et al [23] specifically tailored for Israeli climate, i.e. winter (December 7-March 30), summer (May 31-September 22), autumn (September 23-December 6) and spring (March 31-May 30, respectively). To assess seasonality as a possible modifier of temperature, we classified the pregnancies as "warm" and "cold". “Warm” pregnancies were those with >50% of gestation occurred during the spring-summer period. While “cold” pregnancies were those where >50% of gestation occurred during the fall and winter.

### Data collection and outcomes

All data were fully anonymized before we accessed them and the ethics committee waived the requirement for informed consent. Clinical data and socio-economic status (SES) were collected from the maternal medical records. The SES was defined by the Israeli Statistical Bureau as a neighborhood level measure on a scale 0–20, after accounting for education level, employment status and other characteristics in each of statistical areas in Israel [19].

Outcomes were defined by diagnosis of preeclampsia in pregnancy, based on the following codes of the International Classification and Disease, Ninth Revision (ICD 9): mild or unspecified pre-eclampsia (642.4), severe pre-eclampsia (642.5) and eclampsia (642.6).

### Statistical analysis

In a univariate analysis, we described maternal, pregnancy and fetus’s characteristics, in pregnancies with and without preeclampsia. Additionally, we described the average temperatures of all pregnancies by seasons and trimesters, and the percent of pregnancy weeks occurring in each season. Continuous normally distributed variables were described by means and standard deviations (SD), and analyzed using independent t-test, continuous variables deviating from normal distribution or ordinal variables were reported as medians and interquartile ranges (IQR) and analyzed using Mann-Whitney tests; categorical variables were described by frequencies and proportion out of available cases, and analyzed using Chi square tests. Significance levels below 0.05 were considered significant.

To illustrate the association between temperature exposure during the pregnancy and preeclampsia prevalence, we presented the locally estimated scatterplot smoothing (LOESS) curves [24] of the percent of pregnancies with preeclampsia diagnosis, out of the overall count of pregnancies exposed to the same averaged temperature. We stratified the analysis by trimesters and by "warm" and "cold" pregnancies, to assess the possibility of effect modification.

We conducted a generalized estimating equation (GEE) Poisson multivariable regression models to assess the association between temperature and all preeclampsia pregnancies. We used Pearson dispersion scaling to correct for overdispersion and an exchangeable matrix for covariance within the same cluster formed by deliveries of the same woman. Models included averaged temperatures for each trimester in IQR units (8.9° for 1^st^ and 2^nd^ trimesters and 9.1° for the 3^rd^) as the primary exposure at study and were adjusted to maternal age, gravidity, multiple pregnancies and a history of preeclampsia in past deliveries. We stratified the analysis by ethnicity and by "warm"/"cold" pregnancies to assess possible interactions. Results were reported as relative risks (RR) along with their 95% confidence intervals (95%CI) and p-values. The averaged trimester-specific temperature associations were illustrated in forest-plot figs. The analysis was performed in STATA, version 12 (College Station, TX, StataCorp LP, USA), using the xtgee command.

We further conducted a similar regression analysis for early and late preeclampsia, as they are considered different in their pathophysiological characteristics [25].

### Sensitivity analysis

We used a generalized additive Poisson regression with a random intercept for each woman to assess the dose response curve of the temperature—preeclampsia association. We allowed a nonlinear association by adding a spline function of temperature and plotted the dose response curve. As the curve showed a linear relationship, we treated temperature exposure as linear throughout the analysis.

## Results

### Study population

During the study period, there were 136,547 deliveries, 88,476 (65%) of them were included as they were registered in Clalit HMO and resided in Southern Israel. We then excluded from the analysis pregnancies that did not meet the inclusion criteria and patient's record did not include the exact home addresses, resulting in 64,566 (47.3%) deliveries, delivered by 31,101 women.

Preeclampsia affected 7% (2,173/31,101) of all women and 4.1% (2,617/64,566) of deliveries. Of these, 1,898 (72.5%) were diagnosed with mild preeclampsia, 692 (26.4%) with severe and 27 (1.0%) with eclampsia. Women with preeclampsia were older compared to the rest of the cohort (29.3 ± 6.4 vs. 28.4 ± 5.7, mean ± SD), more likely to be from the Jewish ethnicity group (43.8% vs. 40.8%) and with lower parity [median 2 vs. 3]. Their offspring were more likely to have a low Apgar score and lower birth weight (Table 1). Furthermore, most of the preeclampsia deliveries occurred after weeks 34 and 37 (91.2% and 73.6% respectively), with a mean of 5.5 days from the preeclampsia diagnosis (day of admission) to delivery.

**Table 1:** Maternal and fetus characteristics.

	Characteristics	Preeclampsia (2617, 4%)	Non- Preeclampsia (61949, 96%)	P-value
Maternal	Maternal age, years			<0.001
	Mean±sd (n)	29.3±6.4 (2617)	28.4±5.7 (61931)	
	Median	29.0	28.0	
	Min; Max	18.0; 50.0	18.0; 50.0	
	Jews, % (n/N)	43.8% (1147/2617)	40.8% (25298/61949)	0.003
	SES^1^, median (IQR)	4.0 (3.0–9.0)	4.0 (3.0–9.0)	0.001
Pregnancy	Gestational week,			<0.001
	Mean±sd (n)	37.7±2.7 (2617)	39.0±2.0 (61949)	
	Median	38.0	39.0	
	Min; Max	25.6; 42.0	22.0; 42.0	
	Gravidity,			<0.001
	Mean±sd (n)	3.8±3.3 (2617)	3.9±2.8 (61949)	
	Median	3.0	3.0	
	Min; Max	1.0; 19.0	1.0; 20.0	
	Parity,			<0.001
	Mean+sd (n)	3.2±2.8 (2617)	3.4±2.4 (61949)	
	Median	2.0	3.0	
	Min; Max	1.0; 17.0	1.0; 20.0	
Fetus /newborn	Male Sex, % (n/N)	50.1% (1312/2617)	51.1% (31666/61949)	0.323
	Apgar 1 min<5, % (n/N)	7.0% (180/2581)	2.8% (1703/61048)	<0.001
	Apgar 5 min<7, % (n/N)	2.2% (58/2583)	0.9% (519/61042)	<0.001
	Birth Weight,			
	Mean±sd (n)	2352.5±718.1 (2617)	3156.9±535.3 (61949)	<0.001
	Median	2935.0	3185.0	
	Min; Max	536.0; 4860.0	510.0; 4992.0	
	SGA^2^ of Singleton pregnancies, % (n/N))	17.2% (430/2494)	10.5% (6381/60767(	<0.001

^1^ SES–socio-economic status

^2^ SGA–small to gestational age

### Temperature and seasonality

Table 2 describes the temperatures' summary by seasons and trimesters, and the percent of pregnancy weeks covered by each season. Mean daily temperatures averaged over a trimester were similar; 19.7°± 4.8° in the first trimester, 20.4° ± 4.9° in the second and 20.3°± 4.9° in the third. Averaged seasonal temperatures were different with 26° ± 1.5° and 13.7° ± 2.7° in summer and winter, respectively, vs. 20.1° ± 3.8 and 20.4° ± 2.5° in fall and spring (p-value<0.001).

**Table 2:** Description of temperature by season^1^ during the study period.

Variable	Mean±sd	Median (Min—Max)
Average Daily Temperature in Each Trimester (C°)		
First trimester	19.7±4.8	19.6(15.4–24.2)
Second trimester	20.4±4.9	21.0(16–24.9)
Third trimester	20.3±4.9	20.9(15.8–24.9)
Average Daily Temperature in Each Season (C°)		
Summer	26.0±1.5	20.9(20–31.7)
Winter	13.7±2.7	13.6(5.2–24.5)
Fall	20.1±3.8	20.7(8.9–28.2)
Spring	20.4±2.5	20.4(13.1–27.5)

^1^Seasons were defined according to Alpert et al.: winter (December 7-March 30), summer (May 31-September 22) each lasts about 4 months (3 months and 23 days), autumn (September 23-December 6) and spring (March 31-May 30) each lasts 2 months (75 days and 61 days, respectively).

### Temperature and preeclampsia prevalence

**Fig 1:**
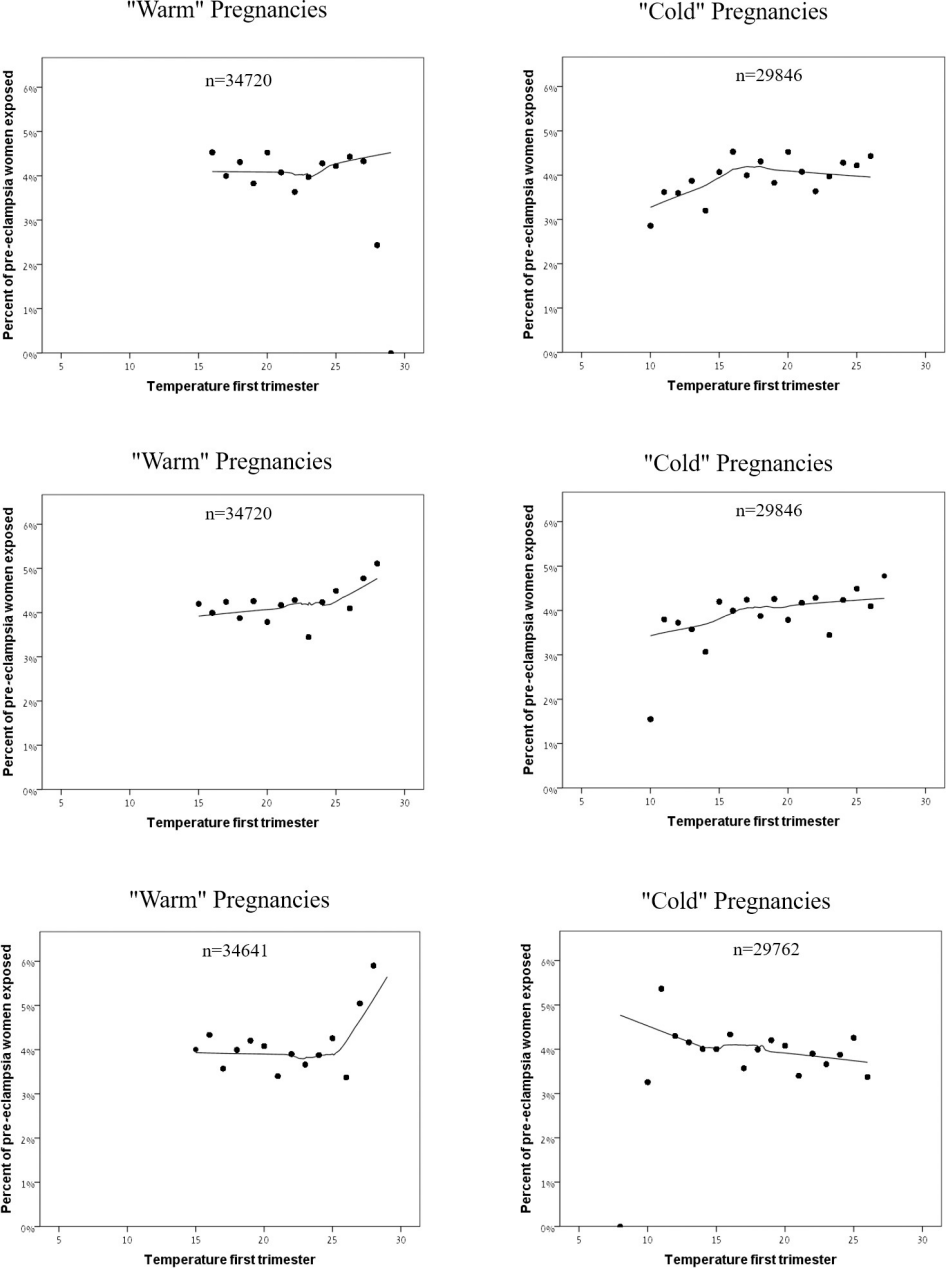
A Preeclampsia Prevalence and Trimester Averaged Temperature in “Warm” vs. “Cold” Pregnancies, 1^st^ Trimester. The LOESS curves of the percent of preeclampsia pregnancies as a function of averaged daily temperatures, out of the “warm” pregnancies vs. the “cold” pregnancies. For instance, out of all women exposed to an average daily temperature of 15° in “cold” pregnancies, approximately +3% were diagnosed with preeclampsia. B Preeclampsia Prevalence and Trimester Averaged Temperature in “Warm” vs. “Cold” Pregnancies, 2^nd^ Trimester. The LOESS curves of the percent of preeclampsia pregnancies as a function of averaged daily temperatures, out of the “warm” pregnancies vs. the “cold” pregnancies. For instance, out of all women exposed to an average daily temperature of 15° in “warm” pregnancies, approximately +4% were diagnosed with preeclampsia. C Preeclampsia Prevalence and Trimester Averaged Temperature in “Warm” vs. “Cold” Pregnancies, 3^rd^ Trimester. The show the LOESS curves of the percent of preeclampsia pregnancies as a function of averaged daily temperatures, out of the “warm” pregnancies vs. the “cold” pregnancies. For instance, out of all women exposed to an average daily temperature of 15° in “warm” pregnancies, approximately 4% were diagnosed with preeclampsia.

Fig 1A–1C describe the association between averaged trimester temperature and preeclampsia incidence. The adverse effect is especially pronounced in “warm” pregnancies, indicating an effect of larger magnitude for high temperatures (>25C°). The positive association between high temperature values and preeclampsia was not that evident in “cold” pregnancies, where the figs indicated either a weak positive link to preeclampsia (first and second trimesters) or a negative association (third trimester).

### Multivariate regression analysis

The overall elevation of one IQR (≈9.0°) of the average temperature during the first trimester for the entire population was associated with a RR = 2.38 (95% CI 1.50; 3.80), a RR = 2.91 (95% CI 1.98; 4.28) for an elevation of one IQR during the second trimester and a RR = 2.91 (95% CI 1.98; 4.28) during the third.

**Fig 2:**
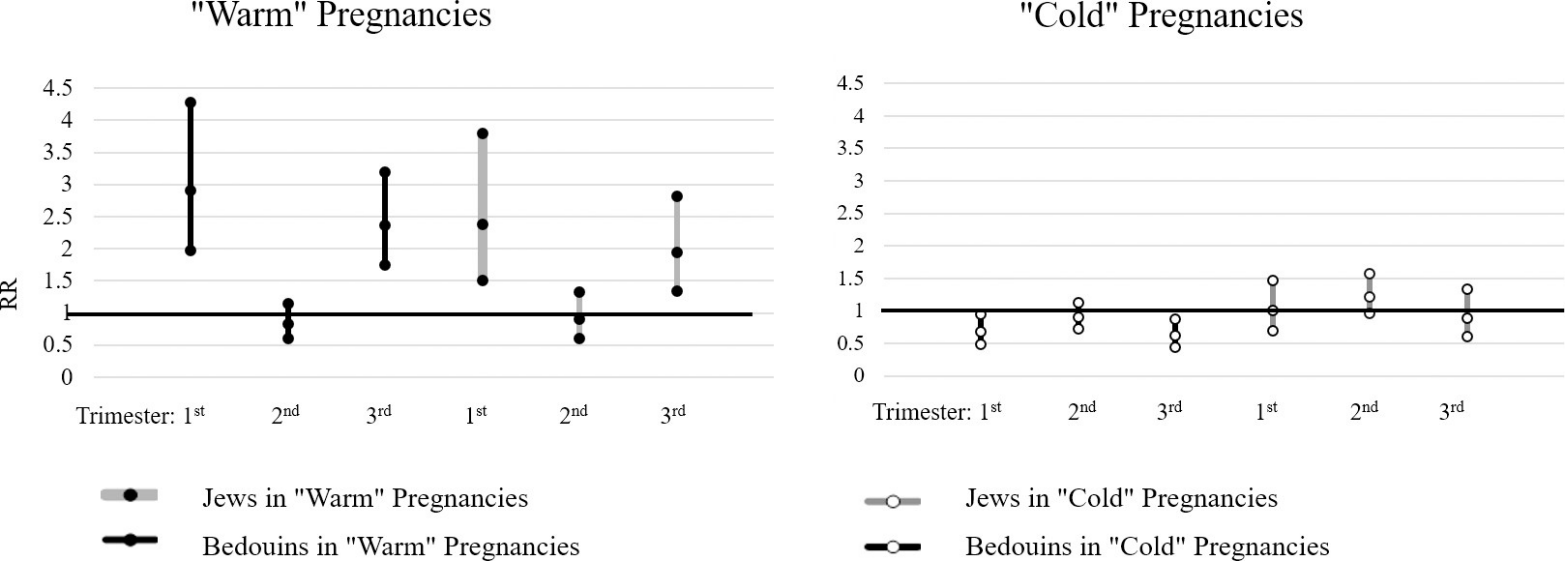
Association between temperature and preeclampsia for “warm” and “cold” pregnancies; multivariable regression models. The association between the trimester-specific temperature and preeclampsia by Jewish and Bedouin-Arab ethnicity, in "warm" and "cold" pregnancies. The Y axis represents the RR of the multivariable models, and the X axis the trimesters. The models included averaged temperatures for each trimester in IQR units as the primary exposure at study and were adjusted to maternal age, gravidity, multiple delivery and a history of preeclampsia in the past deliveries. Results in the tables are presented by the relative risks (RR), their 95% confidence intervals (CI) and p-values.

Fig 2 and Table 3 demonstrate the association between the averaged IQR temperature in each trimester and preeclampsia, stratified by ethnicity and “warm”/”cold” pregnancies. In “warm” pregnancies, an elevation of one IQR (≈9.0°) of the average temperature during the first trimester was associated with a RR = 2.38 (95% CI 1.50–3.80) among women of Jewish ethnicity, and 2.91 (95% CI 1.98–4.28) among those of Bedouin-Arab ethnicity to develop preeclampsia. A similar change in average temperature during the 3rd trimester of “warm” pregnancies was associated with a RR = 1.94 (95% CI 1.34–2.81) and 2.37 (95% CI 1.75–3.20) among women of Jewish and Bedouin-Arab ethnicity, respectively. In “cold” pregnancies, an elevation of one IQR of average temperature was associated with a lower risk to develop preeclampsia among patients of Bedouin-Arab ethnicity RR = 0.68 (95% CI 0.49–0.94) for the 1^st^ trimester and RR = 0.62 (95% CI 0.44–0.87) for the 3^rd^ trimester.

**Table 3:** Multivariable regression models, stratified by ethnicity and “warm”/”cold” pregnancies.

	All Pregnancies (n = 64394)			“Warm” Pregnancies						“Cold” Pregnancies					
				Bedouin-Arab ethnicity (n = 12293)			Jewish ethnicity (n = 14079)			Bedouin-Arab ethnicity (n = 20559)			Jewish ethnicity (n = 17463)		
Effect of temperature in:	P-value	RR	CI	P-value	RR	CI	P-value	RR	CI	P-value	RR	CI	P-value	RR	CI
Trimester 1	<0.001	1.36	1.15–1.61	<0.001	2.91	1.98–4.28	<0.001	2.38	1.50–3.80	0.021	0.68	0.49–0.94	0.974	1.01	0.69–1.47
Trimester 2	0.006	1.10	1.03–1.19	0.257	0.83	0.60–1.15	0.587	0.90	0.61–1.33	0.371	0.90	0.73–1.13	0.109	1.22	0.96–1.57
Trimester 3	0.004	1.28	1.08–1.51	<0.001	2.37	1.75–3.2	<0.001	1.94	1.34–2.81	0.007	0.62	0.44–0.87	0.582	0.89	0.60–1.34

Results of the GEE Poisson multivariable regression models, with PET development as an outcome. The models included averaged temperatures for each trimester in IQR units as the primary exposure at study and were adjusted to maternal age, gravidity, multiple delivery and a history of preeclampsia in the past deliveries. Results are presented by the relative risks (RR), their 95% confidence intervals (CI) and p-values.

When analyzing the effect of temperature on the risk to develop early preeclampsia, we found that during “warm” pregnancies: 1) an elevation of one IQR (≈9.0°) in the average temperature during the first trimester was associated with an increased risk for the subsequent development of early preeclampsia, i.e. RR = 111.45 (95% CI 27.62–449.66), and RR = 436.7 (95% CI 89.14–2139.88) in women of Jewish and Bedouin-Arab ethnicity, respectively; and 2) A similar change in average temperature during the 3rd trimester was associated with increased risk for early preeclampsia RR = 24.18 (95% CI 7.69–75.98) and RR = 155.17 (95% CI 42.32–568.91) among patients of Jewish and Bedouin-Arab ethnicity, respectively.

During “cold” pregnancies, an elevation of one IQR of average temperature during the 1^st^ and 3^rd^ trimesters was associated with a lower risk to develop early preeclampsia both among those of Jewish ethnicity [1^st^ trimester- RR = 0.08 (95% CI 0.03–0.27); 3^rd^ trimester—RR = 0.12 (95% CI 0.03–0.39)] and women of Bedouin-Arab ethnicity [1^st^ trimester- RR = 0.06 (95% CI 0.02–0.17); and, 3^rd^ trimester—RR = 0.06 (95% CI 0.02–0.147)] (Table 4).

**Table 4:** Association between temperature and early and late preeclampsia, stratified by ethnicity and “warm”/”cold” pregnancies; multivariable regression models.

	Bedouin-Arab ethnicity						Jewish ethnicity					
a. Early PE	"Cold" Pregnancies			"Warm" Pregnancies			"Cold" Pregnancies			"Warm" Pregnancies		
Variable	PV	RR	CI	PV	RR	CI	PV	RR	CI	PV	RR	CI
Temperature trimester 1	<0.001	0.06	0.02–0.18	<0.001	436.7	89–2139	<0.001	0.08	0.03–0.27	<0.001	111.45	27.6–450
Temperature trimester 2	0.27	0.67	0.32–1.4	0.01	0.2	0.06–0.69	0.59	1.2	0.57–2.7	0.003	0.18	0.06–0.55
Temperature trimester 3	<0.001	0.06	0.02–0.17	<0.001	155.2	42–569	0.001	0.12	0.03–0.39	<0.001	24.2	7.7–76
b. Late PE												
Variable	PV	RR	CI	PV	RR	CI	PV	RR	CI	PV	RR	CI
Temperature trimester 1	0.58	0.91	0.64–1.29	0.02	1.62	1.08–2.45	0.19	1.03	0.88–1.98	0.27	1.33	0.8–2.2
Temperature trimester 2	0.59	0.94	0.74–1.18	0.95	0.99	0.7–1.4	0.15	1.02	0.93–1.57	0.23	1.3	0.85–1.98
Temperature trimester 3	0.31	0.82	0.57–1.2	0.02	1.48	1.07–2.04	0.65	1.01	0.72–1.7	0.12	1.38	0.92–2.03

Results of the GEE Poisson multivariable regression models, with early and late preeclampsia as an outcome. The models included averaged temperatures for each trimester in IQR units as the primary exposure at study and were adjusted to maternal age, gravidity, multiple delivery and a history of preeclampsia in the past deliveries. Results are presented by the relative risks (RR), their 95% confidence intervals (CI) and p-values.

### Principal findings

Principle findings of the study: 1) higher temperatures are associated with an increased likelihood for preeclampsia when most of the pregnancy occurred during a warm season and this association is stronger among rural-nomadic (women of Bedouin-Arab ethnicity) than those who live in urban environment (women of Jewish ethnicity); 2) among rural-nomadic women, during the cold seasons the upper temperature IQR had a protective effect against the development of preeclampsia; and 3) the effects of temperature on the risk to develop preeclampsia were increased among patients who developed early onset disease.

### Clinical implications - temperature and preeclampsia

Our study is the first to determine the association between the changes in temperature during the entire length of gestation and the subsequent development of preeclampsia. This is in contrast to previous reports that mostly studied the association between this syndrome and the seasons at conception and/or delivery [11, 14–17, 26]. The impact found for temperature in the 1^st^ and 3^rd^ trimesters’, indicates a possible role for the burden of heat at the beginning and end of gestation on maternal physiology that increases the risk for preeclampsia.

The mechanism by which higher temperatures during warm weather predispose a mother to preeclampsia is still under investigation. A possible explanation for such association between the high temperature at the beginning of pregnancy and the probability to subsequently develop preeclampsia can be related to the impaired placental bed [27–29], derived from the study of Higazi et al [30], who tested the effect of temperature on the migration of human trophoblast cells in culture plate. The authors reported that in temperatures at a physiologic range trophoblastic cells gain a symmetric distribution at the warmer edge of the culture plate due to migration of the cells. Lower temperatures were associated with abnormal migration of these cells. This observation is in accord with a previous publication by our group that low temperatures at the beginning of pregnancy are a risk factor for a small for gestational age neonate [22]. Moreover, Higazi et al reported that in high temperature the systemic distribution of the cells was disrupted as well, and the authors suggested that “the capacity of trophoblasts to migrate in response to small differences of temperature within the physiologic range may contribute to the initiations of placental development before the contact with maternal circulation" [30]. Thus, high temperature may affect blastocyst implantation and placentation leading to the subsequent development of preeclampsia.

Our observation that exposure to warm temperatures during the third trimester increases the risk for preeclampsia can possibly be explained by the metabolic burden inflicted by elevated temperatures on maternal physiology that compromise her ability to cope with the physiologic demands of the developing fetus [31, 32] (graphical abstract 1+2). Indeed, during the third trimester there is a decrease in the physiologic function of the placenta that is reflected in a decreased angiogenic placental growth factor (PlGF) [33–40] and a partial O_2_ pressure in the inter-villous space [41] along with substantial fetal growth that occurs during this period. Collectively, these processes lead to a trophoblastic stress and increased secretion of antiangiogenic factor soluble Vascular Endothelial Growth Factor Receptor (VEGFR) -1 by the trophoblast. When the ratio between PlGF and s-VEGFR-1 reaches a critical point, the mother may develop preeclampsia [33–39]. We propose that the increased burden of high temperature on maternal physiology could lead to the aggravation of the trophoblastic stress and the subsequent development of preeclampsia.

### Clinical implications–modification by ethnicity

The effect of high temperature on the subsequent risk for the development of preeclampsia in women of Bedouin-Arab ethnicity was of higher magnitude than that observed for patients of Jewish ethnicity, for both, "warm" and "cold" pregnancies. This observation suggests that women of Bedouin-Arab ethnicity have a higher susceptibility to ambient exposure, that may be related to their nomadic habitant and the conditions in their households frequently lacking electricity and other facilities that may assist in coping with the burden of high temperatures during a warm weather and low temperatures during the cold season. While most Jewish households are equipped by an air conditioner, around 40% of patients with Bedouin-Arab ethnicity are living in temporary localities and only 22% of them [42] use air conditioners. Based on our findings, we would like to propose that air conditioning may mitigate the adverse effects of temperature fluctuations during warm and cold weather, as it may decrease the maternal investment in temperature homeostasis. This assumption is supported by studies [43–45] showing a declining pattern of the effect of temperature on mortality, related to improved technologies, such as air conditioners [46].

Additionally, our observation may be relevant to the effect of global warming. The burden of climate change on health affecting the most unprotected layers in the population has been well established by others [47–49]. In addition, we present here evidence that global warming may have an effect on human reproduction and maternal and/or fetal/neonatal morbidity and mortality. Indeed, according to our findings, in nomadic or low-income societies that lack the weather balancing effect of air conditioning, the burden of elevated temperature may predispose the mothers to preeclampsia. This understanding is also important from an economic perspective [50, 51].

### Strengths and limitations

The study benefited from the temperature estimates individually verified for each participant and obtained based on the satellite spatiotemporally 1 X 1 km resolution model. Additionally, the population-based approach, by which we accounted for most of the deliveries in the region, minimizes the possibility of a selection bias in the study population.

The study has few limitations. Although, the exposure estimates for each mother were assigned by her geocoded home address, we could not account for the time she spent at her home address. Likewise, the maternal indoor exposure was not measured. This might have caused a certain misclassification of the exposure assessment, which must have attained our conclusions towards a null hypothesis. With that been said, we were able to record statistically significant associations, in spite of the possible misclassification.

We could not assign an address to about 25% of the women in the study population. While this proportion corresponds to the rate of unknown addresses of the Central Bureau of Statistics in Israel, we have to assume that our study population excludes citizens with untraceable records and they are frequently of Bedouin-Arab origin.

Furthermore, our investigation is limited to the area of southern Israel, a semi-arid region featured by relatively high temperatures and low humidity. Therefore, our findings can be generalized onto other regions only after accounting for specific meteorological conditions in the area.

## Conclusions

In conclusion, extreme temperatures weather high during warm and low during cold seasons are associated with an increased risk for the development of preeclampsia, and mostly in nomadic society (of Bedouin-Arab ethnicity). These findings suggest that environmental factors such as temperature affect maternal heat homeostasis that may result in reallocation of maternal resources from the developing fetus and may increase the risk for preeclampsia.

Our observation is especially relevant in the context of global warming and its effects on maternal reproductive health. As the Negev area in the southern part of Israel is featured by a semi-arid climate, frequently subjected to high temperatures reaching 42°C during the summer. Therefore, our finding may reflect the future reproductive consequences of global warming.

## Supporting information

S1 File Clinical definitions. (DOCX)

S1 Graphical Abstract (TIF)

S2 Graphical Abstract (TIF)
