# Supplementary material for: Temperature and preeclampsia: Epidemiological evidence that perturbation in maternal heat homeostasis affects pregnancy outcome
Source: PLoS One. 2020 May 18;15(5):e0232877. doi: 10.1371/journal.pone.0232877 (PMC7234374; doi:10.1371/journal.pone.0232877)
Supplement: S1 File — (DOCX) [file pone.0232877.s001.docx]

***Preeclampsia*** was defined as new-onset hypertension after 20 weeks of gestation (systolic or diastolic blood pressure ≥140 mm Hg and/or ≥90 mm Hg, respectively, measured on at least two occasions, 4 hours to 1 week apart) accompanied by proteinuria (≥300 mg in a 24-hour urine collection, or two random urine specimens obtained 4 hours to 1 week apart of ≥1+ by dipstick or one dipstick ≥2+ protein). Early preeclampsia was defined as pregnancies with preeclampsia diagnosis and a delivery before week 34 and late preeclampsia as a pregnancy with preeclampsia diagnosis and a delivery after week 35, according to Li XL et al [24].

***Small for gestational age*** (SGA) – birthweight < the 10th percentile and **L*arge for gestational age (***LGA) – birthweight > the 90th percentile according to regional growth curves [25].

Pathologic Apgar score was defined as <5 at 1 minute and <7 at 5 minutes.

**Perinatal mortality** was defined as either: 1) stillbirth including both antepartum death (APD) and intrapartum death (IPD); and 2) post-partum death (PPD) defined as any neonatal death that occurred within the first 28 days after delivery.
