# Supplementary figures and images for: Temperature and preeclampsia: Epidemiological evidence that perturbation in maternal heat homeostasis affects pregnancy outcome
Source: PLoS One. 2020 May 18;15(5):e0232877. doi: 10.1371/journal.pone.0232877 (PMC7234374; doi:10.1371/journal.pone.0232877)

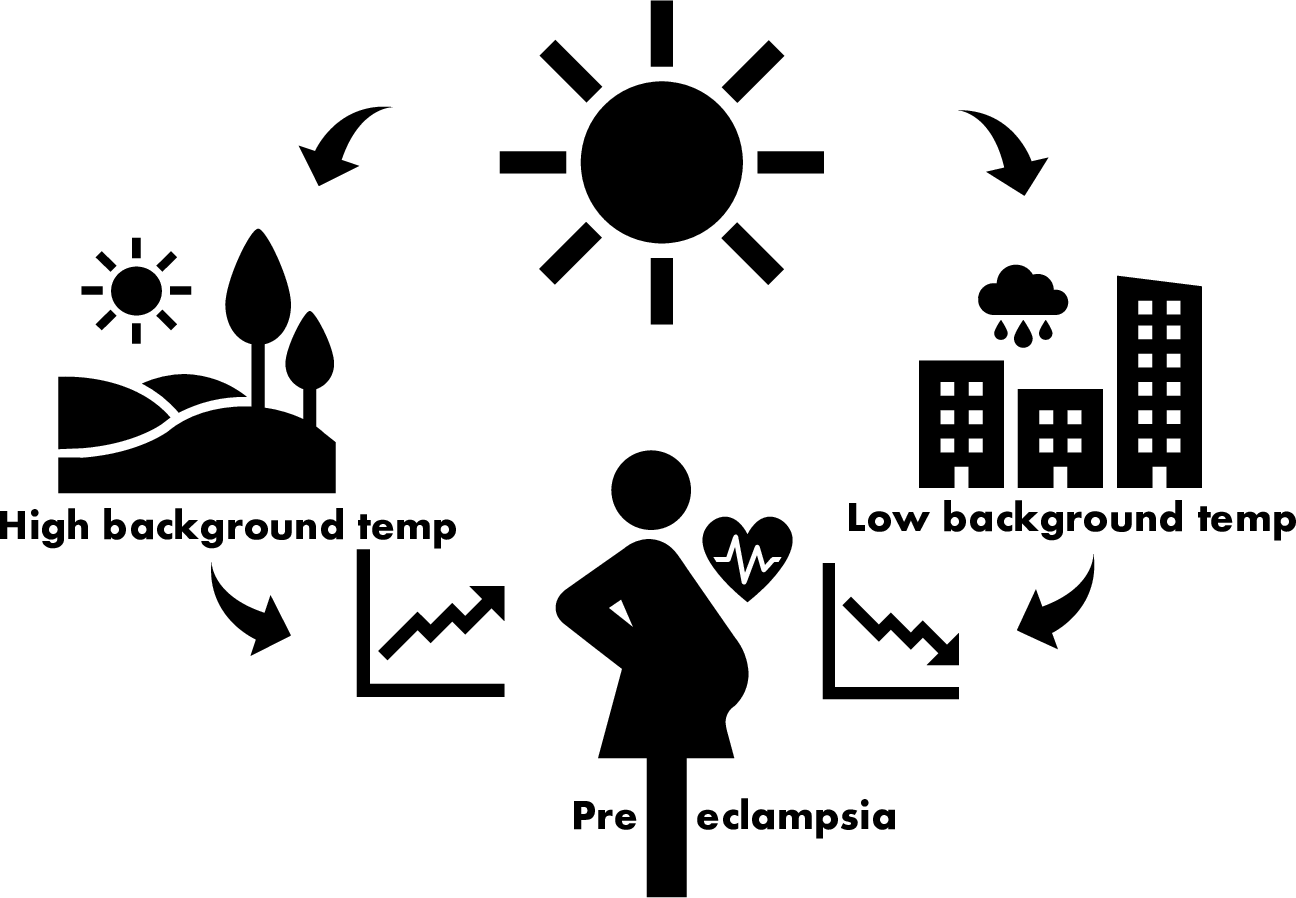

Supplement: S1 Graphical Abstract — (TIF) [file pone.0232877.s002.tif]

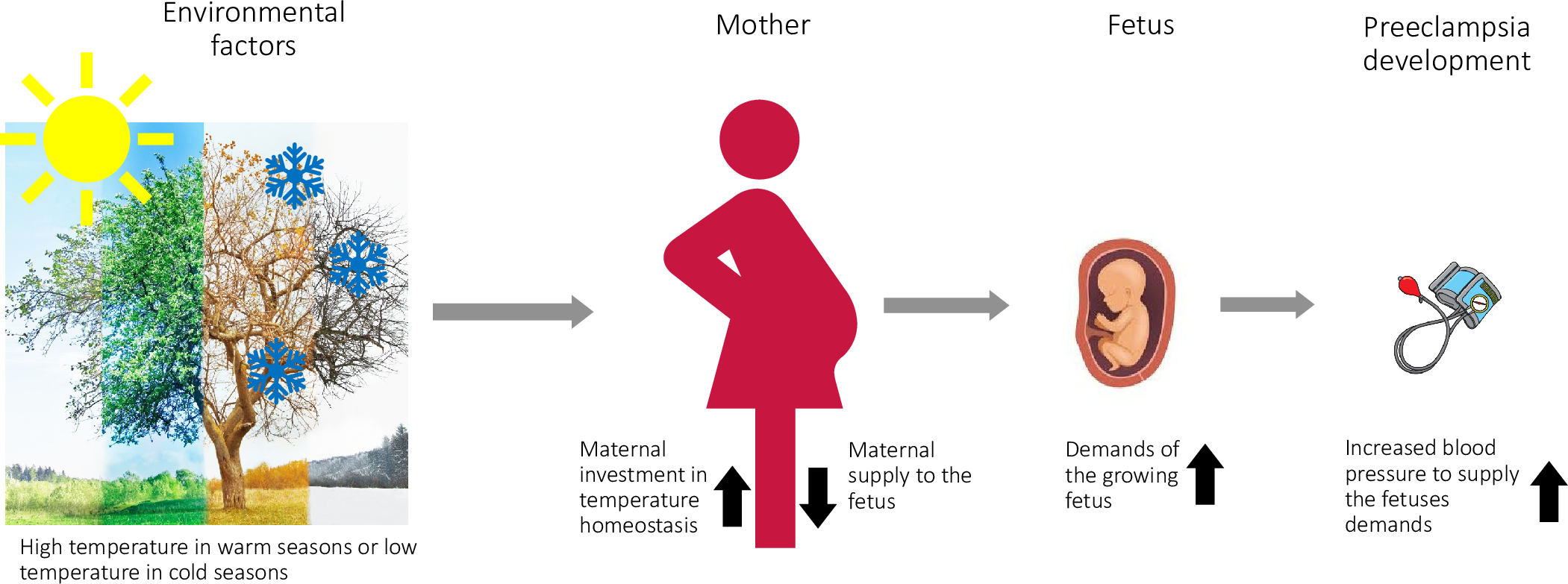

Supplement: S2 Graphical Abstract — (TIF) [file pone.0232877.s003.tif]
